# Cytomegalovirus Reinfections Stimulate CD8 T-Memory Inflation

**DOI:** 10.1371/journal.pone.0167097

**Published:** 2016-11-21

**Authors:** Joanne Trgovcich, Michelle Kincaid, Alicia Thomas, Marion Griessl, Peter Zimmerman, Varun Dwivedi, Valerie Bergdall, Paul Klenerman, Charles H. Cook

**Affiliations:** 1 Department of Surgery, The Ohio State University, Columbus, OH 43210, United States of America; 2 Department of Surgery, Beth Israel Deaconess Medical Center / Harvard Medical School, Boston MA 02215, United States of America; 3 College of Veterinary Medicine, The Ohio State University, Columbus, OH 43210, United States of America; 4 Department of Medicine, Oxford University, Oxford, UK; University of St Andrews, UNITED KINGDOM

## Abstract

Cytomegalovirus (CMV) has been shown to induce large populations of CD8 T-effector memory cells that unlike central memory persist in large quantities following infection, a phenomenon commonly termed “memory inflation”. Although murine models to date have shown very large and persistent CMV-specific T-cell expansions following infection, there is considerable variability in CMV-specific T-memory responses in humans. Historically such memory inflation in humans has been assumed a consequence of reactivation events during the life of the host. Because basic information about CMV infection/re-infection and reactivation in immune competent humans is not available, we used a murine model to test how primary infection, reinfection, and reactivation stimuli influence memory inflation. We show that low titer infections induce “partial” memory inflation of both mCMV specific CD8 T-cells and antibody. We show further that reinfection with different strains can boost partial memory inflation. Finally, we show preliminary results suggesting that a single strong reactivation stimulus does not stimulate memory inflation. Altogether, our results suggest that while high titer primary infections can induce memory inflation, reinfections during the life of a host may be more important than previously appreciated.

## Introduction

Generation of immunological memory after infection is a cornerstone of immunity, providing hosts with long-term protection against infectious diseases and providing the basis for all vaccines. Many pathogens induce prompt T-cell responses that initially expand in response to infections, then contract to a small population of persistent central-memory cells. Some pathogens, however, induce large T-cell responses that persist and do not contract, a phenomenon described as “memory inflation”. Such T-memory inflation has been described for several pathogens, including members of the herpes virus family (herpes simplex viruses and cytomegaloviruses), murine polyoma virus, and acute parvovirus B19 [1–4]. Among these, the best studied are the cytomegaloviruses (CMV), ubiquitous pathogens classified within the *Beta herpesvirinae* subfamily.

Fascination with CMV-specific CD8-memory inflation began with its discovery in the mid 80’s [5], and advanced with discovery of T cell epitope specificities and development of major histocompatibility complex (MHC) multimers. Hence, investigators have characterized these large populations of CMV-specific CD8 T-cells in several murine systems [6–15], and numerous studies in humans have confirmed this biologic phenomenon [16–23]. Perhaps most dramatic of these are observations by Sylwester et al, showing that CMV-specific T-cells can account for >20–30% of T-memory cells in some previously infected patients [20].

These studies have also demonstrated incongruity in CMV-induced T-memory inflation between mice and men. It has become evident that unlike most described murine models that develop consistent and unanimous T-memory inflation after CMV infection, not all humans develop such inflation. In fact, many CMV-infected humans seem to show very little inflation, while others mount enormous CMV-specific T-cell responses [16, 17, 20, 23]. Although there are some reports suggesting that development of inflated T-memory may require time, leading to higher incidences in elderly humans [17–19], other work has suggested that such inflation may not change with time [16], or at best correlate only loosely with age [22]. Further, there are several studies that have shown inflated T-cell responses already exist in young people [20–22].

These findings together led us to wonder if memory inflation observed in most murine models is a consequence of the conditions of primary infection. This hypothesis is supported by work from several investigators that suggests that mice infected with very low doses of CMV develop CD8 T-memory responses but not classic memory inflation [15, 24–27]. Complicating matters further, recent human data show that reinfection can occur despite pre-existing “immunity” [28–31], and the role that reinfection plays in memory inflation remains undefined. Finally, most investigators have supposed that memory inflation is a consequence of reactivation events, but this assumption lacks definitive proof.

One shortcoming of human studies to date is that very basic details about primary infections are rarely known in most immune competent humans, much less occurrence of reinfections or reactivation episodes. Without these data it is impossible to decipher the conundrum of variable memory inflation in humans. We therefore utilized a well characterized model of murine CMV (mCMV) to study the contributions of primary infection, reinfection, and reactivation to CMV-specific immune responses. In this report we define how these three factors can influence CD8 memory inflation and antibody responses to CMV.

## Materials and Methods

### Animals

Female BALB/c mice (Harlan, Indianapolis IN) 6–8 weeks of age were housed in an AAALAC-accredited animal facility, isolated from other mice, monitored daily for early removal criteria, adhering to the *Guide for the Care and Use of Laboratory Animals* prepared by the National Research Council (NIH Publication No. 86–23, revised 1985) following approval by Institutional Animal Care and Use Committee of The Ohio State University Office of Responsible Research Practices. Every effort was made to minimize animal suffering and distress. Mice were euthanized by cervical dislocation under isoflurane inhalation anesthesia. Mouse tissues were dissected aseptically and underwent lymphocyte isolation or were snap frozen in liquid nitrogen, then stored at –80°C.

### Viral infection, reinfection, and reactivation stimulation

Purified Smith strain (VR-1399) mCMV (ATCC, Rockville, MD) was passaged once through fibroblasts (3T3 cells, ATCC, Rockville MD). Primary mCMV infection was achieved by intra-peritoneal (i.p.) injection of 10^2^, 10^3^, 10^4^, or 10^6^ plaque forming units (pfu) Smith-mCMV. All mice underwent primary infections at 8–10 weeks of age. For reinfection experiments, mice were infected first with Smith-mCMV at 10^2^ pfu, followed by 10^2^ or 10^6^ pfu of strain K-181 (kind gift Bret Wing—Shenk lab). For transcriptional reactivation stimulation, 1μg/gm lipopolysaccharide (LPS) from *Escherichia coli* 055:B5 (Sigma) was administered i.p.

### Quantification of infectious virus via plaque assay

Mouse organs were dissected aseptically, ground through a 100μM strainer and re-suspended in 2ml supplemented DMEM. A 1:10 dilution series of the organ lysates was prepared and kept on ice. 48-well plates containing 70% confluent MEF were infected with 100μl of each titration. Centrifugal enhancement of infectivity was applied at 650 x g for 30min at 21°C. The cells were overlaid with 500μl methylcellulose and incubated at 37°C in 5% humidified CO_2_ for 72h-96h until pfu / ml were determined.

### qPCR for quantification of viral and host genes

Mouse tissues were homogenized using a TissueLyser (QIAGEN GmbH, Hilden, Germany) per manufacturer instructions. DNA were isolated with DNeasy Blood & Tissue Kits (QIAGEN GmbH, Hilden, Germany), eluted in 100μl 10mM Tris-HCl, 0.5mM EDTA (pH 9.0) and stored at –20°C. The following primers/probes were used for quantification of mCMV ***gB (M55)***: gB-for2: 5'-GAGAACTGCGACACGAACAG-3'; gB-rev2: 5'-AGCACCTTGAAGTCGGTGTT-3'; gB-p1: 5'-CGATGTCCAGCCCGA TCAGG-3' (GeneBank acc. no. GU305914.1). For the diploid cellular gene ***pthrp***: pthrp-for1: 5´-CAAGGGCAAGTCCATCCAAG-3´; pthrp-rev1: 5´-GGGACACCTCCGAGG TAGCT-3´; pthrp-p1: 5´-TTGCGCCGCCGTTTCTTCCTC-3´ (GeneBank acc. no. NM_008970.3). DNA were amplified in a total volume of 20μl with 1μM of each primer and 250μM of probe using the TaqMan® Fast Universal PCR Master Mix (Catalog no: 4366072, Applied Biosystems, Carlsbad, CA). qPCR were performed on a StepOnePlus Real-Time PCR system (Applied Biosystems, Carlsbad, CA) with the following cycling conditions: 95°C for 20 seconds followed by 45 cycles of 95°C for 1 second and a combined annealing/extension step at 60°C for 20 seconds, during which data were collected. Absolute quantification of viral genes was performed using a plasmid containing the sequences of *gB* and *pthrp* as previously described [32].

### Antibody detection

Blinded sera were evaluated by Charles River Research Animal Diagnostic Services (Wilmington, MA) for mCMV reactive IgG antibody by enzyme-linked immunosorbent assay ELISA. Briefly, this assay uses proteins from infected cell lysates coated on Luminex beads and an anti-mouse IgG secondary antibody (not sub-type specific).

### Identification of mCMV-specific T-cells by flow cytometry

Lymphocytes were isolated from blood and lungs as previously described [9]. For sequential monitoring experiments blood samples were obtained by superficial temporal vein puncture. Fluorescent dye-conjugated antibodies specific for CD8a (PerCP) and CD44 (APC) (BD PharMingen, San Diego, CA) and MHC-I tetramers specific for mCMV proteins m123/pp89 (H2L^d^-restricted ^168^YPHFMPTNL^176^) and m164 (H2D^d^-restricted ^257^AGPPRYSRI^265^) were used to identify mCMV-specific CD8 T-cells as previously described [9]. For phenotyping experiments, antibodies for CD8a (APC) (BD PharMingen, San Diego, CA), CD27 (Pacific Blue) KLRG1 (FITC) CD44 (Percpcy5.5), and CD62L (APCCy7) (all from Biolegend, San Diego CA) were used. MHC class I peptide tetrameric complexes were assembled with phycoerythrein (PE) conjugated streptavidin as previously described [9]. Lymphocytes were incubated with tetramers for one hour (37°C) followed by antibody surface staining for 30 minutes (4°C), fixed, and analyzed by flow cytometry (FACScalibur, Becton Dickinson, Mountain View, CA or LSR II BD Biosciences, San Jose, CA) and results analyzed using FlowJo software (Tree Star Inc., Ashland, OR). To control for run to run variability in tetramer staining in longitudinally monitored cohorts, tetramer-specific CD8 T-cells are presented relative to non-treated high titer (10^6^ pfu) infected age matched controls.

### Statistical Analyses

Statistical analyses were performed using two tailed Students t-test where appropriate. p-values < 0.05 were considered significant for all testing. Means are expressed as mean ± standard error. Statistical software used was GraphPad Prism 5 (GraphPad Software Inc., La Jolla, CA).

## Results

### mCMV-specific T-cell responses are virus dose-dependent

**Fig 1:**
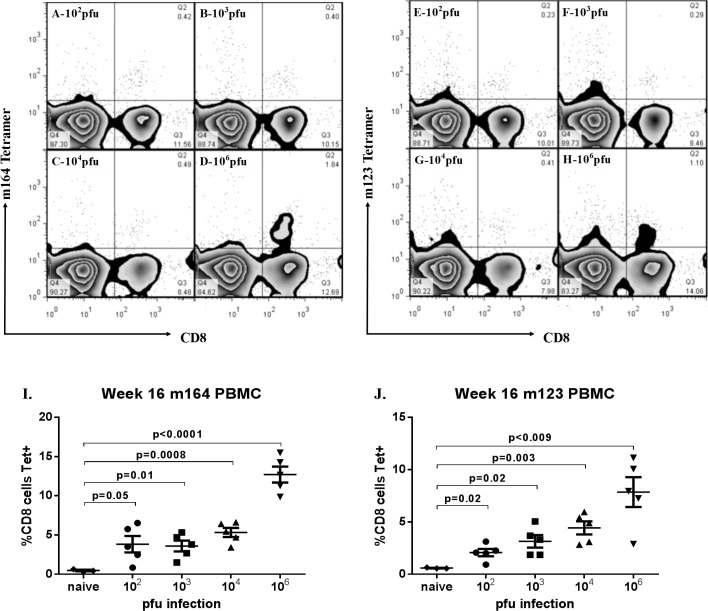
Murine Cytomegalovirus (mCMV) specific T-cell inflation depends upon infecting dose. Mice 8–10 weeks old infected intraperitoneally with 10^2^ (**A&E**), 10^3^ (**B&F**), 10^4^ (**C&G**), or 10^6^ (**D&H**) plaque forming units (pfu) Smith strain mCMV had peripheral blood mononuclear cells (PBMC) evaluated 16 weeks after infection for inflationary mCMV-specific T-cells. PBMC incubated with tetramers specific for m164 (**A-D**) or m123 (**E-H**) epitopes and CD8 were evaluated by flow cytometry and representative scatter plots from individual mice are shown. Mice infected with 10^6^ pfu mCMV show classic inflation of m164 and m123-specific CD8+ cells as previously described by others (**D&H**). In contrast, mice infected with lower titers show less inflation by 16 weeks (m164 **A-C**, pp89 **E-G**). **I&J.** Summary of m164 and m123-specific T-cell results for each cohort 16 weeks after infection. Results represent a single longitudinal experiment. For I-J each point represents a single mouse, and bars represent mean and standard error for each cohort with corresponding p-values in comparison to naive.

**Fig 2:**
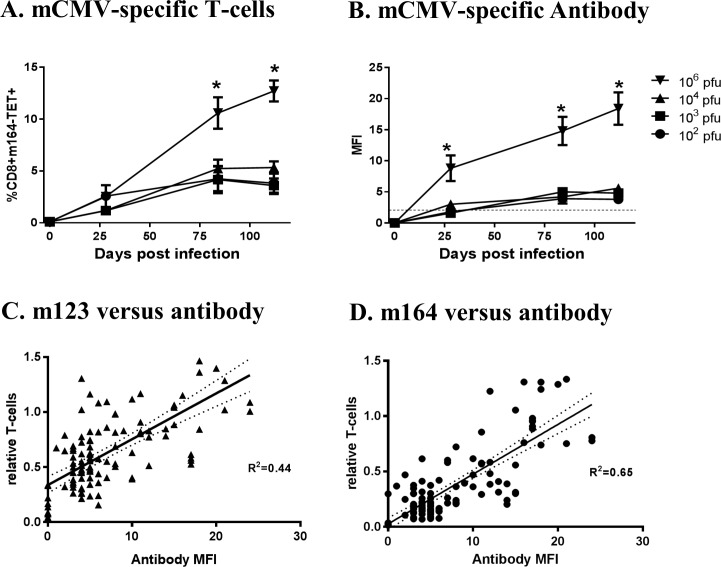
Inflation of murine cytomegalovirus (mCMV) specific T-cells and antibody over time. Mice 8–10 weeks old infected intraperitoneally with 10^2^, 10^3^, 10^4^, or 10^6^ plaque forming units Smith mCMV had peripheral blood obtained longitudinally 4, 12 and 16 weeks after infection evaluated for mCMV-specific T-cells and antibody. **A.** m164-specific CD8+ T-cells were enumerated by flow cytometry using tetramers. **B.** Sera were evaluated for mCMV specific IgG by ELISA with results reported in mean fluorescent intensities;—indicates the limit of sensitivity of the assay. For **A&B**, * indicates significantly higher than all lower titer cohorts (P<0.05). Results represent a single longitudinal experiment with each point/bar representing the mean and standard error for n = 5 mice.

CD8 T-cell responses are known to be critical to mCMV control during acute infection and latency. Recent work has suggested that lower doses of mCMV during primary infection influence the absolute magnitude of the CD8 T-cell response [24, 27], but not the early expansion/contraction of mCMV-specific T-cells nor early development of T-memory [15]. What remains unclear is whether low titer viral infections will stimulate long term T-cell inflation similar to high titer infection. We hypothesized that mCMV-specific T-cell expansion may be viral dose dependent. To test this, we evaluated mCMV specific T-cell responses after infections with 10^2^−10^6^ pfu Smith mCMV. Peripheral blood mononuclear cells were evaluated by flow cytometry 4, 12 and 16 weeks after infection for m123 and m164-epitope specific CD8 T-cells. CMV-specific T-cell responses are expressed as percentages of all T-cells (and not numerically) to make them comparable to published results from humans. As shown in **Fig 1**, mice receiving 10^6^ pfu mCMV developed “classic” inflation of both m164 and m123 specific CD8 T-cells by 16 weeks after infection. In contrast, lower titer infections (10^2^−10^4^) induced significantly diminished expansion of mCMV-specific T-cell responses compared to high titer infections (**Fig 1A–1J**). CD8 T-cell responses in the 10^2^−10^4^ pfu groups were not significantly different from each other at any time point (analysis not shown). Lungs 16 weeks after infection showed an identical pattern of m123 and m164-specific CD8 T-cell responses to infection when compared with peripheral blood mononuclear cells (PBMCs, not shown). The time courses of T-memory responses were similar for both m123 and m164 specific CD8 T-cells, with summarized responses to epitope m164 for each cohort plotted in **Fig 2A.** For both m123 (not shown) and m164-specific **(Fig 2A)** T-cells, there was divergence between low and high titer groups becoming significant by 12 weeks after infection.

### Infecting mCMV inoculum determines magnitude of antibody response

We have previously observed that vivarium housed mice can develop very low level mCMV infections without measurable antibody responses [24]. Similarly, there are reports of humans who are hCMV DNA positive by PCR assay but have no detectable hCMV specific antibody [33, 34]. In our murine model, these “occult” infections require highly sensitive nested-PCR methods to detect viral DNA, suggesting that these infections are likely very low titer. We therefore hypothesized that the magnitude of in-vivo antibody responses following mCMV infection might be predicated by the initial infection titer similar to T-cell responses. To test this hypothesis, mice infected with 10^2^−10^6^ pfu Smith mCMV by intraperitoneal injection had serial serum evaluations for mCMV-specific antibody by ELISA. As illustrated in **Fig 2B**, high titer infections with 10^6^ pfu (10^5^−10^6^ pfu typically used in published mCMV models) induce robust IgG antibody responses detectable 4 weeks after infection that increase by week 16. In contrast, low titer infections induce antibody responses either at or just above the limit of detection (MFI ≤ 2) early after infection (4 weeks). Low titer infections ultimately develop detectable humoral responses, but they remain significantly lower than high titer infections with 10^6^ pfu. These data suggest that similar to T-cell responses, the humoral response to CMV infections is highly dependent upon the magnitude of the initial infection, and that high titer infections can induce CMV-specific “antibody inflation”. In fact, when CMV-specific T-cell and antibody are compared in the same mouse, there is excellent correlation (**Fig 2C & 2D**).

### Viral load in tissues

**Fig 3:**
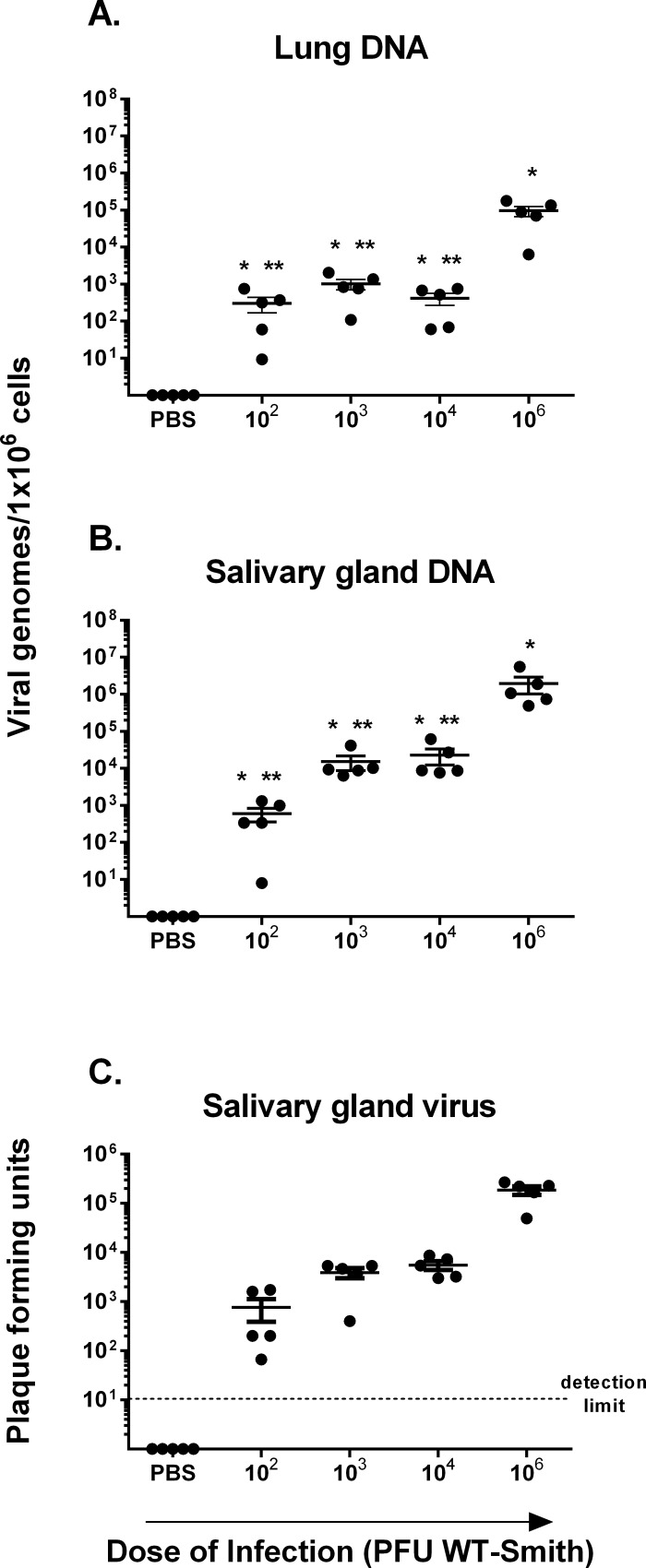
Verification of productive infection and viral DNA load of mice infected with different doses of mCMV Smith. Mice infected at 8–10 weeks of age with 10^2^, 10^3^, 10^4^ or 10^6^ plaque forming units (pfu) mCMV Smith strain or mock treated with PBS were evaluated on day 10 post infection. Viral DNA loads were determined individually for **A.** lungs and **B.** Salivary glands of 5 mice per group. **C.** Salivary gland viral titers were quantitated in pfu/ml to confirm productive infection. Results represent a single experiment with mean values and standard errors indicated by horizontal bar and error bars, with individual dots representing each mouse. Mock treated mice had no detectable viral DNA or infectious virus. * indicates that means were significantly higher than mock. ** indicates that means are significantly lower than mice infected with 10^6^ pfu. Comparisons were by Students t-test using p-value <0.05.

Recent work by Reddehase et al has suggested that CD8 memory inflation after mCMV infection might be directly related to viral load [26]. If this is true, then based upon our T-cell results, we would expect to see significantly lower viral loads in mice infected with lower titers. To confirm that all mice had developed latent viral infection and further to determine viral load, we performed both qualitative and quantitative real-time PCR on tissues from each mouse. Qualitative PCR of DNA from lung homogenates confirmed detectable mCMV-GB DNA in all mice (not shown). Quantitative real-time PCR for lung DNA shows significantly higher viral load after high titer infection than with any of the low titer infections (**Fig 3A & 3B**), a pattern nearly identical to that seen for mCMV-specific T-cells. To confirm that low titer infections are “productive”, we tested salivary gland 1 week after infection for infectious virus. As shown in **Fig 3C**, live virus is detectable in salivary glands of all mice 1 week after infection, regardless of infectious dose. Lung DNA loads from mice 16 weeks after low titer infections show identical patterns with all low titer mice having significantly lower DNA loads than high titer mice (data not shown). Thus mCMV-specific T-cell responses appear to correlate with tissue viral loads after variable titer infections.

### Phenotype of partial memory responses

**Fig 4:**
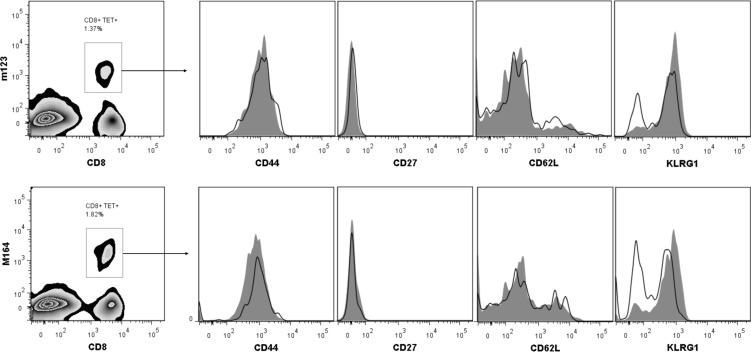
Phenotypic analysis of murine cytomegalovirus (mCMV) specific T-cells after low titer infection. Mice infected intraperitoneally at 8–10 weeks of age with 10^2^ or 10^6^ plaque forming units (pfu) Smith mCMV had peripheral blood evaluated by flow cytometry for m123 and m164-specific CD8 T-cells 16 weeks after infection. mCMV-specific CD8+ T-cells identified with tetramer staining were further evaluated for surface expression of CD44, CD27, CD62L, and KLRG1. Representative superimposed histograms from n = 5 replicates each of low titer (10^2^ pfu, open line) and high titer (10^6^ pfu, gray filled) mice are shown.

To confirm that that low titer infections induce “partial” inflation of CD8 T_EM_, and not merely central T-memory responses, we compared the CD8 phenotype from mice undergoing low titer and high titer infections. mCMV-specific CD8 T_EM_ have been phenotypically characterized as CD44^+^CD62L^lo^CD27^-^KLRG1^+^ cells [8, 9]. Thus PBMC from mice infected with 10^2^ or 10^6^ pfu Smith strain were evaluated 42 weeks after infection for expression of CD44, CD27, CD62L, and KLRG1 on tetramer positive mCMV-specific CD8 T-cells. Mice infected with low titer mCMV show a phenotype consistent with T_EM_ with nearly identical patterns of CD44, CD27, and CD62L as those infected with high titers (**Fig 4**, group p-values all >0.05). However, for mice infected with low titer mCMV there is significantly lower expression of KLRG1 on both m123+ T-cells (68% v. 85%, p = 0.01) and m164+ T-cells (45% v. 74%, p = 0.02). We therefore conclude that low titer infections induce partial inflation of virus-specific T_EM_ cells, and that KLRG1 expression of these cells is diminished relative to T_EM_ developing after high titer infections.

### Influence of subsequent infection upon pre-existing T-cell immunity

Humans, non-human primates, and mice have all been shown to be susceptible to re-infection with CMV [29–31, 35–39]. Because CMV-specific T-memory is protective against lethal infection when adoptively transferred to naïve recipients [10, 13] we were interested to determine how pre-existing memory might influence subsequent memory responses to secondary infection with a different CMV strain.

**Fig 5:**
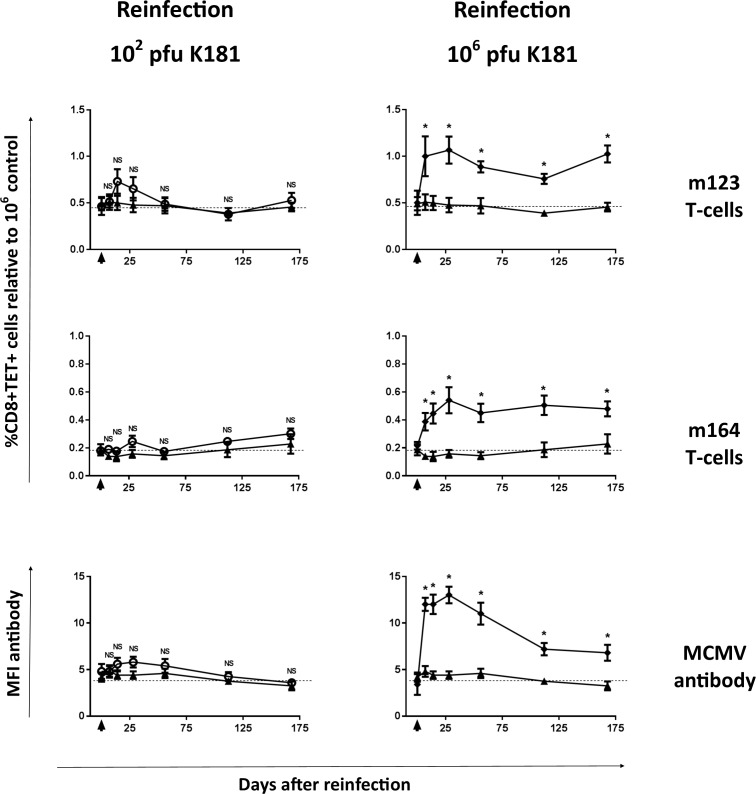
Influence of reinfection on T-cell and antibody inflation. Cohorts of mice infected at 8–10 weeks of age with 10^2^ plaque forming units (pfu) Smith strain murine cytomegalovirus (mCMV) were evaluated over time beginning 154 days (22 weeks) after primary infection. Peripheral blood mononuclear cells (PBMC) were evaluated for m123 and m164-specific T-cells 1 week prior to and then sequentially after reinfection (dark arrowhead) with K181 mCMV or mock treatment with vehicle. CD8 T-cell results following reinfections are expressed relative to age and infection duration matched 10^6^ control mice (not shown). Lower panels show mCMV-specific IgG responses. ▲represents mock reinfection, while ○ and ♦ represent reinfections with K181 at respectively 10^2^ or 10^6^ pfu. Data presented are from single longitudinal experiments with each data point representing means of n = 5 mice, with bars showing standard errors. * indicates significantly different than mock reinfected mice (p<0.05), and NS indicates no significant difference (p>0.05).

To test this, mice latently infected with low-titer Smith mCMV (10^2^ pfu) were re-infected 22 weeks after their original infection. To mimic reinfection with a different strain, mice received 10^2^ or 10^6^ pfu of mCMV K181. Control mice received saline instead of second infections. Peripheral blood was monitored serially for mCMV-specific T-cell and antibody responses. As shown in **Fig 5**, low-titer mice secondarily infected with low titer K181 mCMV (10^2^ pfu) show transient increases in m123 and m164-specific T-cells that quickly return to partially inflated baseline levels. In contrast, low-titer mice secondarily infected with high titer K181 mCMV (10^6^ pfu) show further inflation of both m123 and m164-specific T-cells that persists after the second infection. CD8 T-cell results are expressed relative to age and infection duration matched 10^6^ control mice (not shown). The lower level KLRG1 expression seen on virus specific T-cells after low titer infections (Fig 4) was not significantly increased by reinfection with high titer K181 (data not shown).

Similar results are seen for mCMV antibody, with high titer second infections inducing antibody inflation, while low titer second infections do not induce antibody inflation (**Fig 5**). Interestingly memory T-cells and antibody levels showed excellent correlation (p<0.0001, not shown), suggesting that memory inflation of both T-cells and antibody may be similarly driven by viral load. We attempted to correlate viral load with these immune responses at the end of these longitudinal experiments, however viral loads were too low to allow reliable detection with quantitative PCR (data not shown). Taken together, these results show that reinfection can significantly contribute to memory inflation, and that this is dependent on the re-infecting titer. Low titer re-infections may not necessarily overcome pre-existing immunity to induce further T-cell or antibody inflation.

### Transcriptional reactivation as a trigger of memory inflation

**Fig 6:**
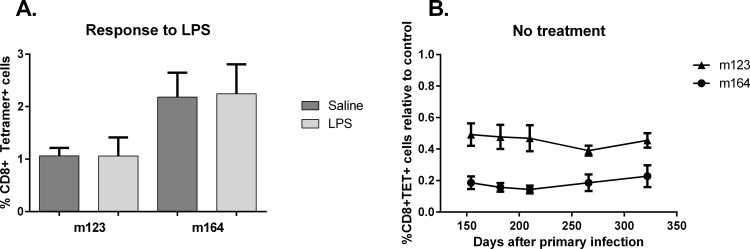
Memory inflation after reactivation stimuli. Mice latently infected with low titer (10^2^ plaque forming units (pfu)) Smith strain cytomegalovirus (mCMV) (infected at 8–10 weeks of age and allowed 16 weeks to develop latency) were treated with a transcriptional reactivation stimulus lipopolysaccharide (LPS) or control (saline) (both administered intraperitoneal (i.p.)). **A**. Latently infected mice (16 weeks post-infection (pi)) were treated with LPS and 12 weeks later peripheral blood mononuclear cells (PBMC) were evaluated for mCMV m123 and m164 specific T-cells. PBMC results are absolute percentages of CD8+ cells binding tetramers. **B**. Latently infected mice 22 weeks after low titer infection had PBMC monitored serially for m123 and m164 specific T-cells, and results are expressed as % of CD8+ cells binding tetramer relative to a 10^6^ pfu control cohort for each time. Data presented are from one of three separate experiments with each point/bars representing means and standard errors from n = 5 mice.

One historically popular explanation of memory inflation in humans has been the contribution of reactivation episodes to memory inflation [40–43]. To test this hypothesis, we used mice latently infected with low titer (10^2^ pfu) Smith mCMV. After confirmation of partial memory inflation 16–20 weeks after infection, mice were treated with sub-lethal LPS, a reactivation stimulus previously shown to consistently cause transcriptional reactivation, [44, 45] or saline control. LPS stimulation did not cause any significant difference in MCMV-specific T-cells (**Fig 6A**). This experiment was performed 3 times, and even 12 weeks post-LPS treatment (shown) there was no significant change in m123/m164 specific T-cells. Likewise, following LPS stimulation there was no significant difference in lung viral load (not shown). Similar to the low titer reinfection controls, mice infected with low titer mCMV that were untreated did not develop memory inflation, even after almost 1 year (**Fig 6B**). These data also show that memory inflation does not develop spontaneously over time in low titer mice. Altogether these results suggest that treatment with an accepted reactivation trigger (LPS) is insufficient to stimulate memory inflation.

## Discussion

This study confirms that the primary infection viral load can drive CD8 T-memory inflation, but more importantly that subsequent reinfections that occur during the life of the host can also contribute to memory inflation. We also confirm the occurrence of antibody inflation [27], and show excellent correlation between antibody and T-memory inflation. Our data suggest that both T-memory and antibody inflation are likely a consequence of tissue viral load. Somewhat surprisingly, our limited results do not support the age old hypothesis that reactivation causes memory inflation. Together these findings may help to explain the broad and inconsistent CMV-specific CD8 T-memory and antibody responses seen in human hosts [16–22, 46, 47].

The concept that CD8 memory inflation can be determined at the time of primary infection and in fact be predicated by the infecting viral load was suggested to us by our earlier published work [24]. This finding has been recently corroborated by Redeker et al who performed very detailed immunophenotyping using a different C57BL6 model [27]. Their work together with the current report suggests that not all primary CMV infections induce full memory inflation, with low titer exposures causing what we have termed “partial” CD8 T-memory inflation. We have chosen this nomenclature for these less inflated responses based on their demonstrated phenotypic similarity to fully inflated memory, but also because such responses are still inflated relative to classic T-central memory responses that contract and become barely measurable 7–10 days after priming [48, 49]. Importantly, partial inflation has been demonstrated in very young CMV-infected humans, whose CMV-specific CD8 T-cells develop T_EM_ phenotypes but are not as inflated as elderly hosts [50], as well as adults whose responses (despite absolute magnitude) seem to be mostly T_EM_. Our findings are also consistent with murine work by others, including Andrews et al. who showed the influence of viral load on magnitude of very early CD-8 T-memory responses in BALB mice [25], Bohm et al who showed correlation between tissue viral load and CD8 inflation in BALB mice [26], and finally work from Snyder et al who have shown similar dose dependent impacts on CD8 memory inflation in C57BL6 mice [15].

Unlike most murine models of CMV infection, where mice are challenged with non-lethal yet very high titer infections, natural infections almost certainly occur at variable titers. Shellam et al have shown that natural mCMV infections in wild mice induce variable antibody responses and viral loads that in context with the current report are consistent with variable-dose infections [38]. Others have suggested that oropharyngeal infections are more natural and can occur at different titers, but whether such infections induce inflated T-memory remains undescribed [51–54]. Corroborating tissue studies of HCMV load in humans are lacking, but we have recently observed that naturally occurring porcine CMV infections show significant variability in tissue viral loads and antibody titers (Dwivedi et al, manuscript in preparation). It therefore seems likely that primary infections in humans occur with variable viral loads, and that this may in part explain the variability seen in memory inflation in humans. It is important to note that the model of intraperitoneal infection/reinfection used in the current report is significantly different from such “natural” infections, perhaps limiting the generalizability of our findings to humans.

In addition to primary infections, a growing body of data suggests that CMV reinfection/coinfection may occur frequently in immune competent hosts. Such reinfections are suggested by studies that show presence of multiple strains after natural infection in humans and mice [31, 38, 55], and have been confirmed experimentally in mice and rhesus macaques by deliberate reinfection [36, 37, 39]. Furthermore, studies of immune competent women suggest CMV reinfection rates ~10% per year, similar to estimates of rates of primary infection [35]. Although Adler et al have shown that pre-existing immunity from naturally occurring CMV infections confers some protection against subsequent CMV infections in humans [56], this protection is obviously not complete or reinfections would not occur. The current report shows for the first time that reinfections can stimulate further inflation of partially inflated T-memory and antibody responses despite preexisting immunity. At this juncture it is not clear whether this expansion is from already primed cells, or an induction of new responses. Our data also suggest that subsequent CMV encounters must be of adequate magnitude to induce further inflation, as low titer reinfections did not induce further memory inflation. Taken together with previously published work, it seems likely that immune competent hosts repeatedly encounter CMV infections during a lifetime, and that some but not all of these encounters may be significant enough to contribute to further CMV-specific memory inflation.

The idea that CMV reactivation contributes to memory inflation in immune competent hosts has been supposed for many years. Support of this hypothesis comes from studies of transplant and critically ill patients showing that reactivation is associated with expansion of T-effector memory cells [57–59]. Further indirect evidence in immune competent hosts comes from recent work by Beswick et al (mCMV) and Lang et al (murine herpes simplex virus), showing that inflated murine CD8 T-memory can be attenuated by prolonged antiviral therapy [3, 60]. Theoretically, prolonged antiviral therapy prevents repeated reactivation episodes and thus removes ongoing stimuli that maintain inflation. If memory inflation were simply a consequence of reactivation episodes, then it stands to reason that reactivation effects would accumulate over the lifetime of the infected host, leading to memory inflation that would be more prevalent in elderly hosts, and previous literature support that hypothesis [18, 19, 61].

Despite the undeniable appeal of reactivation as a trigger of memory inflation, other available data and the current report suggest that this assumption should be more rigorously studied. First, it is noteworthy that not all members of the *Herpesvirinae* family develop memory inflation comparable to CMV despite their proclivity to reactivation (varicella zoster, Epstein-Barr virus) [62, 63]. Second, reactivation is not prerequisite, as viruses containing mCMV epitopes but incapable of replication have been shown to cause T-memory inflation [15, 64]. Finally, the current report suggests that a single strong reactivation stimulus (LPS) that has been previously shown to cause consistent transcriptional reactivation [44, 45] is insufficient to cause memory inflation.

To suggest that reactivation does not cause memory inflation is a significant departure from current assumptions, and we acknowledge that the presented data are incomplete. LPS endotoxemia is a morbid stimulus encountered by very few hosts during a lifetime, and therefore this may not be the most relevant mechanism to explain memory inflation in humans. Furthermore, our longitudinal cohort study design precluded tissue confirmation of reactivation, leaving the possibility that reactivation was not stimulated in these mice. Complicating this further is the issue of sensitivity of detection: our lab has been repeatedly unsuccessful at detecting reactivation by sepsis or LPS after low titer infections (unpublished), consistent with work from others that has shown “reduced risk of reactivation” with low viral loads [65, 66]. It is interesting that LPS stimulation did not increase tissue viral load in mice with previous low titer infections suggesting that reactivation driven inflation might require increased viral loads. It is therefore possible that these results represent failure to actually trigger reactivation and thereby failure to stimulate memory inflation. Conversely, it is conceivable that memory inflation is driven by initial and subsequent reinfections and perhaps not by reactivation events as currently assumed. Whichever the case, it seems appropriate to test this hypothesis further, although such studies will require development of a model of repeated reactivation and detection after low titer infections.

Increasing data support the relationship between memory inflation and continual antigen encounter by CD8 T-cells. Elegant work by Wherry et al has shown that the magnitude and quality of CD8 T-cell memory responses to most viral infections is dependent upon the amount of viral epitope presented [67], and thereafter presence of antigen is typically not required to maintain central T-memory [68]. In contrast, the inflated T-memory responses induced by mCMV infection depend upon continuous antigen stimulation for maintenance [14]. Such memory decays after being transferred to naïve hosts where antigen stimulation cannot occur or in immune competent hosts treated with prolonged antiviral therapy [14, 60]. Consistent with this, available phenotypic data for inflated CMV-specific T-memory suggest that these T-cells are repeatedly encountering antigen [9, 11, 12, 14, 69–73]. For example, KLRG1 expression on T-memory cells has been associated with repetitive antigen stimulation [73], and CMV-specific T-cells have been shown to express this marker [8, 9]. In the current report, we show that low titer infections result in low tissue viral loads, and it seems logical to conclude that these infections induce less inflation because there is less viral antigen to drive such inflation. Decreased KLRG1 expression on CMV-specific CD-8 T_EM_ following low titer infections further supports this hypothesis. It is interesting to note that despite an increase in memory inflation, reinfection did not significantly increase KLRG1 expression on CMV-specific T-cells. This raises a question about tissue viral loads following reinfection, but unfortunately, tissue viral loads after chronic infection/reinfections and reactivation experiments are not quantifiable with currently available methods (likely due to their very long durations), leaving it unclear what contribution reinfection and reactivation make to eventual tissue viral load.

Our findings have several important clinical implications. It is interesting that elderly patients with expanded T-memory populations have been associated with frailty and possibly immune senescence, including impaired responses to vaccines [17, 47, 74–77]. Likewise, those with the highest CMV-specific IgG titers seem to have higher all-cause mortality than those with low titers [46, 47]. Given that high titer primary infection and reinfection might contribute significantly to these phenomena, strategies to reduce primary or prevent reinfection might be desirable. In addition, there is growing interest in using CMV as a vaccine vector for other diseases [37, 78–81]. Given the widespread prevalence of CMV in the population, many of these vaccines would require reinfection of naturally infected hosts. Our results suggest that choosing proper reinfection-vaccination doses will be critical to induce desirable immunity, and that further work to delineate possible undesirable effects of such reinfection-vaccinations is very much needed.

In conclusion, the current report helps to further resolve the conundrum of variable memory inflation in humans following CMV infection. Inflated CMV-specific T-cell responses in humans are likely induced by myriad combinations of primary infection viral load, and likely re-infections that have occurred since the original infection. Given available data in humans and mice, it seems likely that most hosts initially encounter low titers of virus during primary infection, thereby inducing partially inflated CMV-specific memory responses. During the host’s lifetime, reinfections may contribute to further memory inflation of both T-cell and antibody responses. At this juncture, experimental proof that reactivation contributes to memory inflation is lacking and will require further study.

## Supporting Information

S1 File Dataset for PLOS submission. Minimum dataset for Figs 1–6.
(PZF)

S2 File Flow data for PLOS Fig 1. Flow FCS files for Fig 1A–1H.
(ZIP)

## Abbreviations

CMV: Cytomegalovirus
GB: glycoprotein B
hCMV: human cytomegalovirus
i.p.: intraperitoneal
LPS: lipopolysaccharide
mCMV: murine cytomegalovirus
MFI: mean fluorescence intensity
MHC: major histocompatibility complex
PBMC: peripheral blood mononuclear cells
